# Sustainable synthesis and characterization of high-surface-area activated carbons from walnut and pistachio shell wastes via chemical activation

**DOI:** 10.1038/s41598-026-43746-8

**Published:** 2026-03-09

**Authors:** Ali Ender Kuyucu, Ahmet Selçuk, Yunus Önal, İhsan Alacabey, Kadir Erol

**Affiliations:** 1 Institute of Science, Department of Chemistry, Yüzüncü Yıl University, Van, 65080 Turkey; 2 Faculty of Education, Department of Mathematics And Science Education, Yüzüncü Yıl University, Van, 65080 Turkey; 3 Faculty of Engineering, Department of Chemical Engineering, Inonu University, Malatya, 44280 Turkey; 4 Vocational School of Health Services, Department of Medical Services and Techniques, Mardin Artuklu University, Mardin, 47100 Turkey; 5 Vocational School of Health Services, Department of Environmental Protection Technologies, Hitit University, Çorum, 19030 Turkey

**Keywords:** Activated carbon, Agricultural waste, Characterization, Chemical activation, Surface area, Energy science and technology, Environmental sciences, Materials science

## Abstract

Valorization of agricultural residues into high-performance porous carbons is an effective route to sustainable adsorbents. Here, walnut green outer shell and pistachio pink outer shell were converted into activated carbons via chemical activation using KOH (1:1, 1:2, 1:3) and ZnCl₂ (1:1). Without any pretreatment, precursors were carbonized at 500 °C (1 h, nitrogen atmosphere), followed by activation at 800 °C (KOH, 1 h) or 500 °C (ZnCl₂, 1 h). Textural analyses revealed a strong dependence on both precursor type and activating agent. Walnut-shell carbons reached exceptionally high surface areas of 1028–2347 m² g⁻¹, with the maximum obtained for KOH (1:3), whereas pistachio-shell carbons achieved 788–1324 m² g⁻¹ under the same KOH series. In contrast, ZnCl₂ activation produced markedly lower areas (445–750 m² g⁻¹) and grinding caused only minor changes. FTIR/XRD/SEM/Elemental analyses collectively supported the formation of defect-rich, turbostratic carbon frameworks with well-developed porous morphologies, highlighting walnut shell as a particularly promising precursor for sustainable, high-surface-area activated carbons suitable for adsorption-driven environmental applications.

## Introduction

Recent advances in technology and industrialization have significantly improved human quality of life; however, they have also led to serious environmental concerns, the most critical of which is environmental pollution^1^. This issue transcends national borders and requires global attention, as pollutants released into the air, water, or soil ultimately disrupt ecosystems. A notable example of transboundary pollution is the Chernobyl nuclear disaster, which had far-reaching environmental consequences beyond its country of origin. The degradation of ecological balance, coupled with uncontrolled exploitation of natural resources driven by rapid population growth, has exacerbated environmental problems^2^. Among various forms of pollution, water pollution poses a grave threat due to its direct impact on human health. All forms of pollution eventually reach aquatic systems through mechanisms such as rainfall and erosion, making the preservation and remediation of water resources a global priority^3,4^. As clean water becomes increasingly scarce with growing population demands, effective and affordable treatment strategies are urgently needed.

Water pollution, a subset of environmental pollution, refers to the degradation of water quality caused by chemical, physical, and biological agents. Continued pollution of freshwater resources jeopardizes access to safe drinking water, a fundamental human need^5^. According to the Food and Agriculture Organization (FAO), the global population affected by water scarcity was 12% in 1995 and is projected to rise to 15% by 2025. Similarly, the proportion of the world population facing water shortages is expected to increase from 29% in 1995 to 34% in 2025. Over the years, various techniques have been developed to combat environmental pollution. However, many of these methods have drawbacks, such as high capital and operating costs or the generation of secondary pollutants. Consequently, cost-effective, easy-to-implement, and environmentally benign alternative approaches have attracted interest. Among these, the adsorption technique stands out due to its effectiveness in removing pollutants from both liquid and gas phases^6^.

Adsorption allows for the purification and reuse of contaminated water, thereby mitigating water scarcity. The performance of an adsorption system is primarily governed by the characteristics of the adsorbent material, particularly its surface area and pore structure. Larger surface areas and well-developed porosity enhance pollutant uptake by providing more active sites for adsorption. Therefore, optimizing the textural properties of adsorbents is critical for achieving high removal efficiencies^7–10^.

Activated carbon (AC) is one of the most widely used adsorbents in environmental applications due to its high surface area, large pore volume, and excellent adsorption capacity. With increasing demand for activated carbon, attention has turned to low-cost, renewable, and sustainable raw materials for its production^11,12^. Agricultural wastes, abundant in nature and rich in carbon, offer an ideal alternative. Their use not only reduces production costs but also supports waste management and environmental protection^13^.

Biomass, a renewable energy resource, offers a sustainable alternative to fossil fuels. Unlike fossil fuels such as oil, coal, and natural gas, biomass can be replenished in a relatively short time. During their growth, plant-based biomass sources absorb carbon dioxide, making them carbon-neutral when used as fuel or raw material for activated carbon^14,15^. Moreover, the carbon yield and quality of the resulting activated carbon depend heavily on the type and composition of the biomass used. Biomasses with high carbon content are therefore preferred for such applications. The widespread availability and renewability of biomass make it advantageous over conventional fossil fuels^16^. Since biomass can be cultivated in nearly all geographic regions, countries can reduce their dependence on imported energy and benefit from local energy generation. Biomass cultivation also contributes to combating desertification, mitigating greenhouse gas emissions, and creating employment opportunities, thus offering both environmental and socio-economic benefits^17^. Furthermore, biomass can be converted into solid, liquid, or gaseous fuels, providing versatility unmatched by other renewable energy sources. This multipurpose nature makes biomass a valuable resource in energy and environmental remediation technologies^18,19^.

The choice of biomass precursor is crucial in determining carbon yield, pore development, and structural organization during pyrolysis and chemical activation. Recent studies have shown that lignin-rich agricultural residues favor increased aromatic condensation and the formation of thermally stable carbon frameworks, while biomass rich in cellulose and hemicellulose follow different pathways for devolatilization and pore formation^20^. In this context, walnut green and pistachio pink outer shells were intentionally selected as model precursors due to their distinct lignocellulosic compositions and their widespread availability as agro-industrial waste in Türkiye. Walnut shells, which have a relatively high lignin content, are expected to promote the formation of graphenic domains and hierarchical pores during potassium hydroxide (KOH) activation. Meanwhile, pistachio shells have a unique macromolecular structure that enables systematic comparison of activation efficiency and pore development. To explore this precursor–activation synergy, the raw biomasses were directly carbonized at 500 °C for 1 h under a nitrogen atmosphere (100 mL min⁻¹) with a heating rate of 10 °C min⁻¹, without any pretreatment. The resulting biochars were chemically activated using two different agents: KOH at impregnation ratios of 1:1, 1:2, and 1:3, followed by heating at 800 °C for 1 h; and zinc chloride (ZnCl₂) at a 1:1 ratio, activated at 500 °C for 1 h (including both ground and unground forms). The activated carbons were then characterized for their structural and textural properties and tested for dye removal performance, enabling a systematic evaluation of how precursor type and activation chemistry influence porosity development and adsorption behavior. This comparative approach advances sustainable waste valorization strategies and supports the development of cost-effective, environmentally friendly adsorbents for wastewater treatment.

## Materials and methods

### Materials

**Fig. 1:**
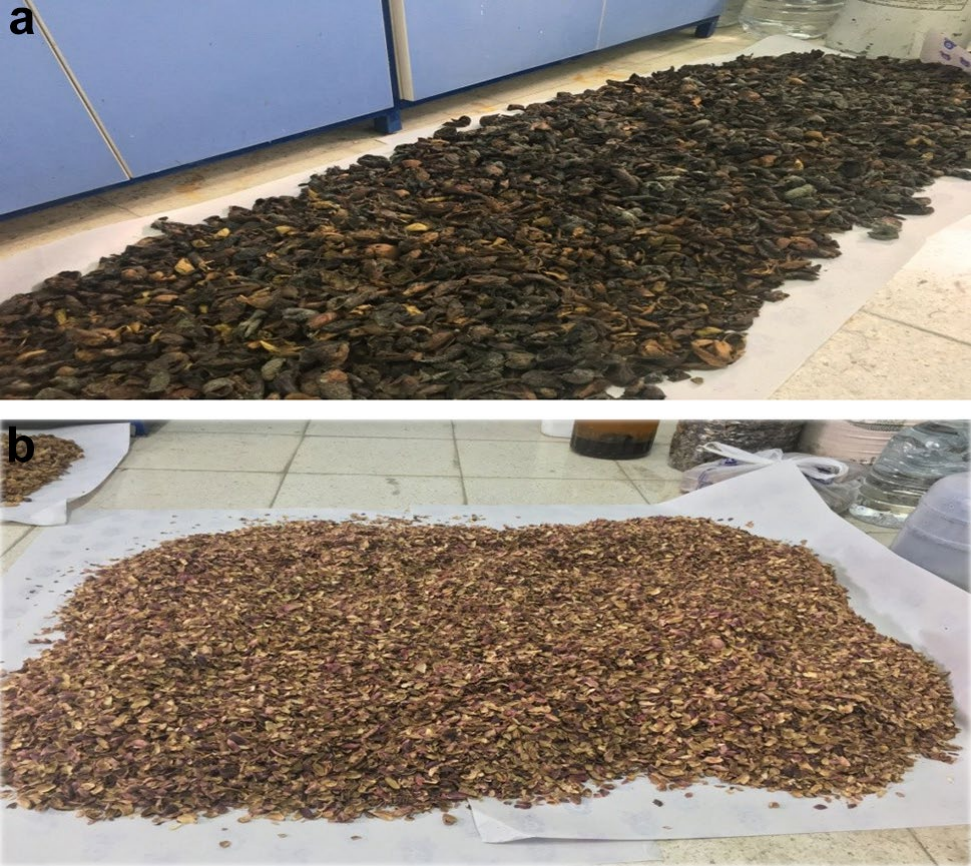
(**a**) Walnut outer shells, (**b**) Pistachio outer shells left to dry in the laboratory.

Walnut green and pistachio pink outer shells were used as biomass precursors in their original form, without any pretreatment. Nitrogen gas was supplied to maintain an inert atmosphere during thermal treatments. Potassium hydroxide (KOH) and zinc chloride (ZnCl₂) were used as chemical activating agents, while dilute hydrochloric acid (HCl) and deionized water were employed for post-activation washing. Carbonization was carried out in a three-zone programmable furnace (Protherm PZF 12/50/700). Fourier Transform Infrared Spectroscopy (FTIR) spectra were recorded using a PerkinElmer Spectrum One spectrometer. X-ray diffraction (XRD) analysis was performed with a Rigaku RadB-DMAX II diffractometer. Surface morphology was examined by scanning electron microscopy (SEM, Leo EV040). Elemental compositions were determined using a CHNS-932 (LECO) analyzer. A reflux condenser system was connected to the furnace outlet to collect condensable liquid products during carbonization. Photographs of the raw biomass materials are shown in Fig. 1.

### Carbonization processes

The raw walnut and pistachio shell samples were directly carbonized without any pretreatment. The process was conducted in a three-zone cylindrical furnace using a steel crucible under a continuous nitrogen flow of 100 mL min⁻¹. Samples were heated at two different heating rates (5 °C min⁻¹ and 10 °C min⁻¹) up to a final temperature of 500 °C and held for 1 h. After completion, the furnace was allowed to cool naturally to room temperature under a nitrogen atmosphere. The resulting biochar samples were collected and stored in airtight containers. The carbonized samples were coded as AFDK3 (5 °C min⁻¹), AFDK8 (10 °C min⁻¹), CDK3 (5 °C min⁻¹), and CDK8 (10 °C min⁻¹).

During carbonization, a reflux condenser was used to collect condensable liquid products. The non-condensable pyrolysis gases were not recycled for energy recovery in this laboratory-scale study and were safely vented under controlled conditions. In industrial-scale operations, these gases can serve as a secondary energy source to enhance comprehensive process sustainability and thermal efficiency.

### Chemical activation and activated carbon preparation

Following carbonization, the biochars were chemically activated with two different activating agents to comparatively evaluate their pore development. Although two different heating rates were examined to evaluate their potential influence on biochar formation, no significant compositional or structural differences were observed. Therefore, the biochars prepared at 10 °C/min were selected for subsequent activation experiments.

#### KOH activation

Carbonized walnut and pistachio shell samples were impregnated with KOH at weight ratios of 1:1, 1:2, and 1:3 using sufficient deionized water to ensure homogeneous mixing. The mixtures were dried at 105 °C and subsequently activated in a tubular reactor at 800 °C for 1 h under nitrogen flow, with a heating rate of 10 °C min⁻¹.

After cooling to room temperature, the activated samples were washed with distilled water, treated with dilute HCl to remove residual inorganic species, and then repeatedly rinsed with distilled water until no chloride ions were detected (silver nitrate test). The products were dried at 105 °C, ground, and stored in sealed containers. These samples were coded AFDK4–AFDK6 and CDK4–CDK6 based on their impregnation ratios.

Although increasing the KOH ratio enhances pore development up to a certain limit, excessively high impregnation ratios may cause structural collapse, excessive burn-off, and reduced carbon yield. Therefore, the KOH ratio was limited to 1:3 to avoid over-activation and ensure structural stability.

#### ZnCl₂ activation

In the second activation method, walnut and pistachio shell samples were impregnated with ZnCl₂ at a 1:1 weight ratio in deionized water. After drying at 105 °C, activation was performed at 500 °C for 1 h under a nitrogen atmosphere with a heating rate of 10 °C min⁻¹.

Following activation, the samples were treated with dilute HCl and thoroughly washed with distilled water until the absence of chloride ions was confirmed. The final products were dried at 105 °C, ground, and stored in sealed containers. These samples were coded as AFDK9, AFDK10, CDK9, and CDK10.

In this study, a ZnCl₂ impregnation ratio of 1:1 was selected as a representative activation condition based on commonly reported literature values. Unlike KOH activation, which strongly depends on impregnation ratio due to its redox-driven pore development mechanism, ZnCl₂ primarily promotes dehydration and cross-linking reactions at moderate temperatures. Therefore, the present work focused on comparative evaluation rather than full optimization of ZnCl₂ activation parameters.

## Results and discussions

### Textural properties and BET analysis

The textural properties of the activated carbons were evaluated to assess the influence of precursor type and activation strategy on surface area development and pore structure (Table 1). For pistachio shell–derived carbons (AFDK series), increasing the KOH impregnation ratio led to a marked increase in specific surface area and mesopore volume. The surface area increased from 788 m² g⁻¹ (AFDK4) to 1324 m² g⁻¹ (AFDK6), accompanied by a substantial rise in mesoporous surface fraction. This trend reflects the intensified chemical etching and lattice expansion induced by KOH at higher ratios, which promotes pore widening and the generation of additional adsorption sites. In contrast, ZnCl₂ activation (AFDK9 and AFDK10) yielded significantly lower surface areas (~ 445–449 m² g⁻¹), indicating limited pore development in pistachio-based carbons under these conditions.

Walnut shell–derived carbons (CDK series) exhibited substantially superior textural properties. The highest BET surface area was obtained for CDK6 (2347 m² g⁻¹), with a dominant mesoporous contribution consistent with reports that walnut-shell-derived activated carbons can reach > 2000 m² g⁻¹ depending on activation strategy and pore development^21^. The larger surface area and pore volume suggest that the walnut shell structure interacts more effectively with KOH, facilitating extensive carbon matrix rearrangement without severe structural collapse. ZnCl₂ activation of walnut shells resulted in moderate surface areas (~ 730–750 m² g⁻¹), again lower than those achieved by KOH. These findings clearly demonstrate that both precursor composition and activation chemistry play critical roles in pore evolution.

The N₂ adsorption–desorption isotherms of the KOH-activated carbons exhibit a combined Type I/IV behavior with a narrow H4-type hysteresis loop according to the IUPAC classification. The steep uptake at low relative pressures confirms the dominance of microporosity, whereas the hysteresis loop at intermediate pressures indicates the presence of slit-shaped mesopores. This hierarchical pore structure is characteristic of biomass-derived activated carbons produced via KOH activation and is consistent with SEM observations (Sect.  3.4) and with the high BET surface areas of CDK5 and CDK6. The exceptionally high surface areas and hierarchical pore structures obtained, particularly for CDK6, are directly relevant for adsorption-driven environmental applications. High micropore volume enhances adsorption capacity for small molecules, while mesopore development improves mass transfer and diffusion kinetics, which are critical parameters in practical wastewater treatment and gas adsorption systems.

**Table 1:** BET results of activated carbons.

Sample	S_BET_ (m²/g)	Smicro (m²/g)	Smeso (m²/g)	V_T_ (cm³/g)	Vmicro (cm³/g)	Vmeso (cm³/g)	d_*p*_ (nm)
AFDK4	788.41	719.98	68.43	0.41	0.38	0.03	2.08
AFDK5	1282.14	1036.28	245.85	0.649	0.564	0.058	2.02
AFDK6	1324.23	937.09	387.14	0.685	0.529	0.11	2.06
AFDK9	449.02	354.91	94.11	0.19	0.12	-	8.66
AFDK10	445.33	350.78	95.63	0.19	0.13	-	8.41
CDK4	1028.27	950.42	77.85	0.54	0.5	0.04	2.12
CDK5	1927.32	708.88	1218.44	1.05	0.5	0.05	2.18
CDK6	2347.4	18.2	2329.19	1.28	0.18	0.19	2.19
CDK9	749.83	455.56	294.27	0.58	0.25	0.25	3.13
CDK10	730.42	459.78	270.63	0.54	0.25	0.27	2.98

d_p_= 4 V/A.

### Pore size distribution

**Fig. 2:**
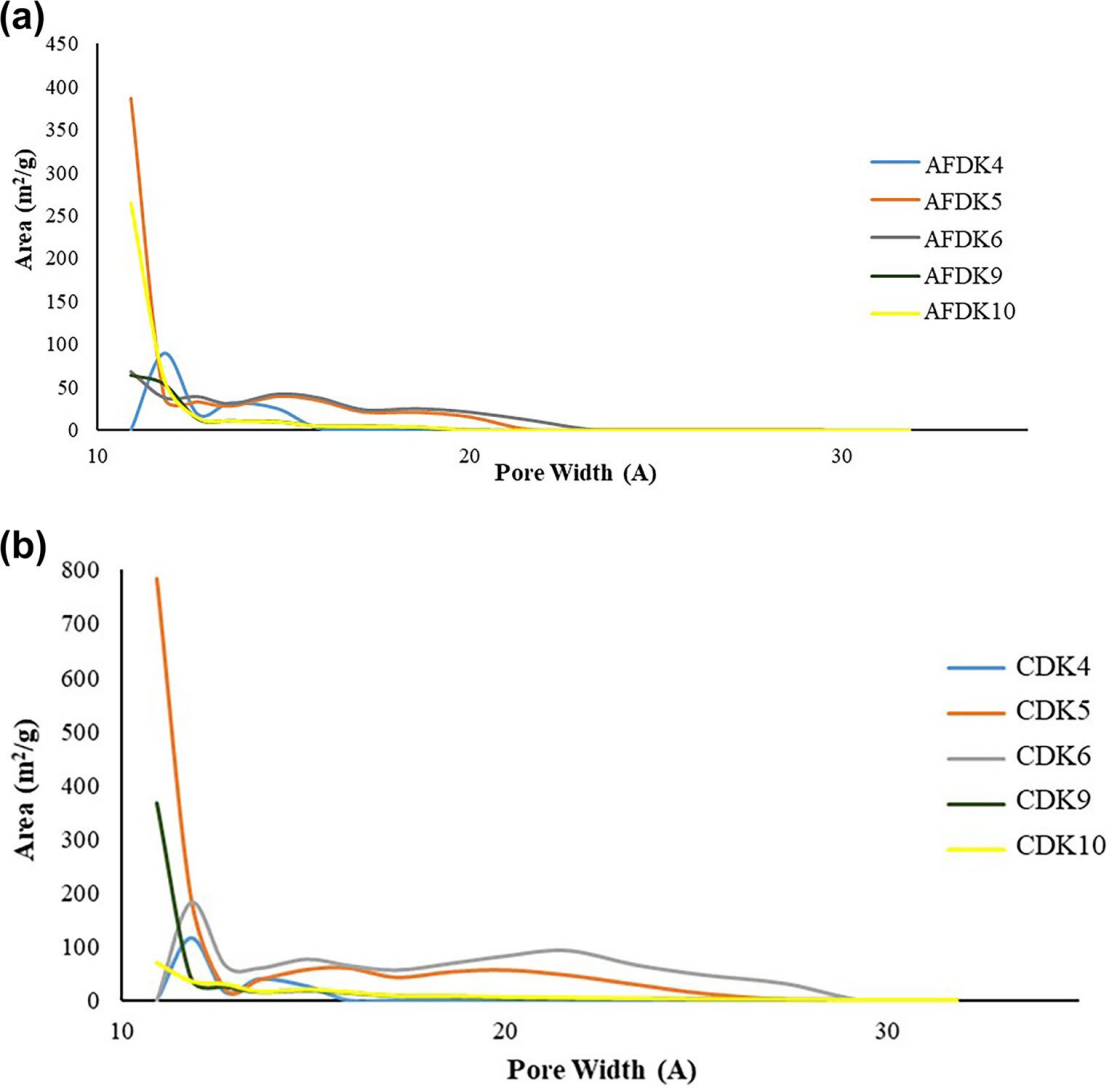
The pore size distribution curves of the (**a**) pistachio and (**b**) walnut shells.

The corresponding pore size distributions, calculated using the BJH and t-plot methods, confirm the development of hierarchical micro–mesoporous structures in KOH-treated carbons, whereas ZnCl₂ activation results in comparatively limited pore widening. The pore size distribution curves obtained from nitrogen adsorption–desorption measurements (Fig. 2) provide deeper insight into the evolution of micro- and mesoporosity as a function of precursor type and activation strategy.

In the pistachio-derived AFDK series (Fig. 2a), a progressive broadening of the mesopore region was observed with increasing KOH impregnation ratio. While AFDK4 exhibited a predominantly microporous structure with limited mesopore contribution, AFDK5 and AFDK6 showed a pronounced increase in mesoporous volume, consistent with their higher BET surface areas. This behavior indicates that higher KOH content not only generates new micropores but also widens existing pores through intensified chemical etching. The pore-widening effect is attributed to redox reactions between KOH and the carbon matrix, which expand the lattice and partially gasify the carbon framework, thereby creating interconnected mesoporous domains^22^.

In contrast, ZnCl₂-activated samples (AFDK9 and AFDK10) displayed a comparatively narrow pore distribution with a dominant contribution in the larger pore region (d_p_ ≈ 8.5 nm), but without significant development of hierarchical porosity. The limited shift in pore size distribution confirms that ZnCl₂ activation was less effective in restructuring the pistachio-based carbon matrix under the applied conditions. This suggests that dehydration and cross-linking mechanisms induced by ZnCl₂ were insufficient to generate extensive pore expansion in this particular biomass precursor^23^.

The walnut shell–derived carbons (Fig. 2b) exhibited a markedly different pore evolution profile^24^. In the CDK series, increasing the KOH ratio led to the development of a hierarchical pore system featuring both microporous and mesoporous contributions. Notably, CDK6 demonstrated a strong mesoporous domain centered around ~ 2–3 nm, which correlates directly with its exceptionally high surface area (2347 m² g⁻¹) and total pore volume (1.28 cm³ g⁻¹). The formation of this hierarchical structure suggests a more favorable interaction between KOH and the walnut shell matrix, enabling controlled pore enlargement without excessive structural collapse^25^.

For ZnCl₂-activated walnut samples (CDK9 and CDK10), the pore size distribution remained relatively similar between ground and unground variants, indicating that mechanical pretreatment exerted minimal influence compared to chemical activation. The mesoporous contribution was moderate but less developed than in KOH-treated samples.

Overall, the pore size distribution analysis clearly demonstrates that KOH activation promotes both micropore generation and mesopore widening, leading to hierarchical porosity, whereas ZnCl₂ primarily induces limited structural rearrangement under the selected conditions. These differences in pore architecture directly account for the observed variations in BET surface area and total pore volume. Besides, the limited differences observed between ground and unground samples indicate that chemical activation is the primary factor controlling pore development under the applied conditions, while mechanical pretreatment exerts only a secondary influence.

### FTIR analysis

**Fig. 3:**
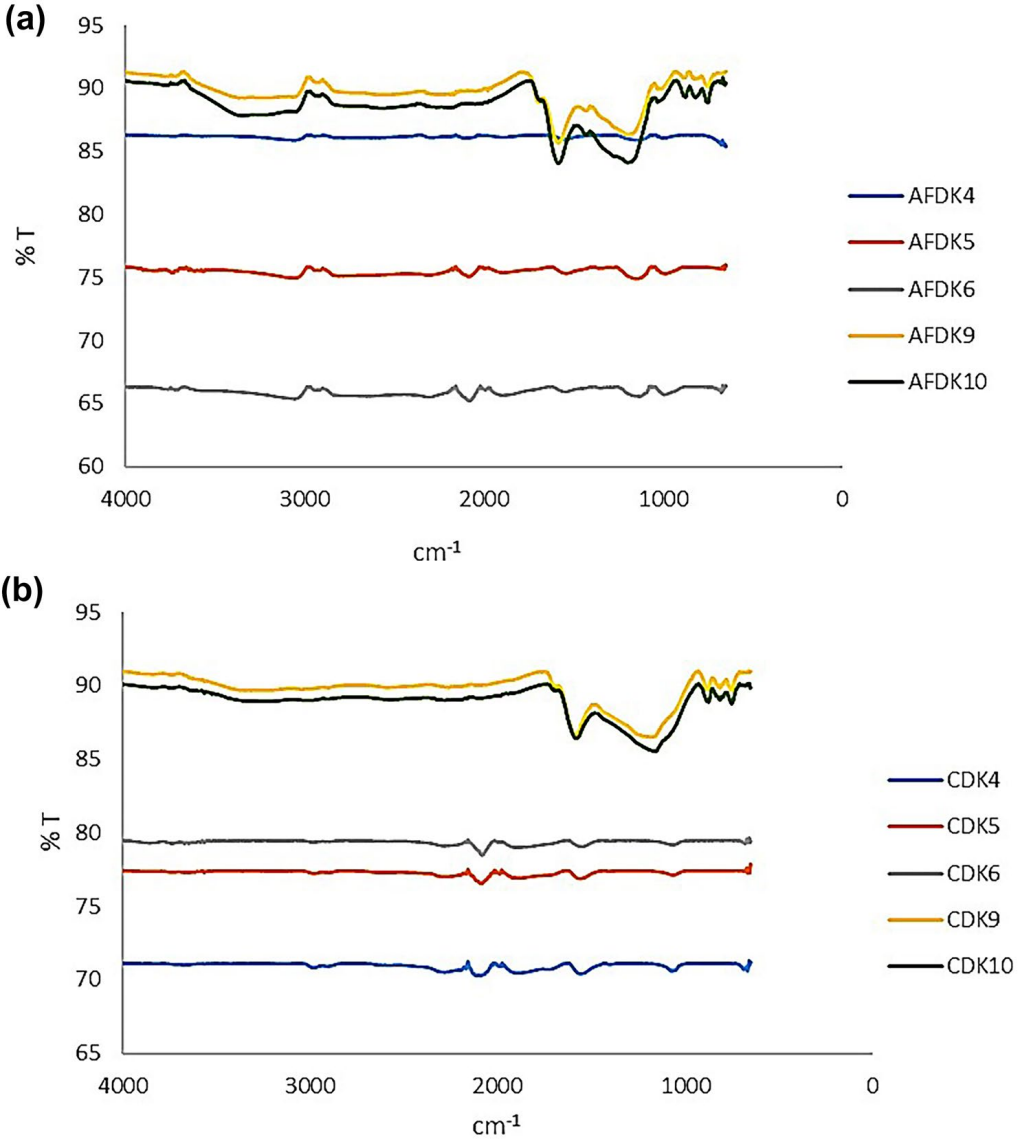
FTIR spectra of activated carbons obtained from (**a**) pistachio and (**b**) walnut shells.

The FTIR spectra of the AFDK samples are presented in Fig. 3a. All samples exhibit similar structural features characteristic of KOH-activated carbons. With increasing KOH ratio, the intensity of functional group-related bands becomes more pronounced^26^. The absorption bands observed around 3100 cm⁻¹, 2100 cm⁻¹, 1575 cm⁻¹, and 1030 cm⁻¹ correspond to the stretching vibrations of O–H, C = O, C = C, and C–O groups, respectively^27^.

The relatively low intensity of these bands suggests that oxygen-containing functional groups are primarily located at the edges of graphite- or graphene-like domains rather than uniformly distributed within the carbon matrix. Increasing the KOH ratio promotes chemical activation and surface oxidation, thereby increasing the abundance of oxygen-containing functionalities^28^. For ZnCl₂-activated samples, the characteristic structural vibrations become comparatively more distinct. The peaks at 1030 cm⁻¹ and 1575 cm⁻¹ are attributed to C–O and C = C stretching vibrations, respectively^29^, while the broad band around 3300 cm⁻¹ corresponds to O–H stretching. The persistence of this broad O–H band indicates incomplete graphitization or partial conversion to graphene-like structures. During ZnCl₂ activation, the biomass undergoes structural rearrangement through dehydration and cross-linking reactions, resulting in a more rigid carbon framework.

The FTIR spectra of the CDK samples (Fig. 3b) exhibit comparable structural features, confirming that KOH activation yields chemically similar carbon frameworks. As the KOH ratio increases, the bands associated with surface functional groups become more evident^30^. The absorption bands near 3100 cm⁻¹, 2100 cm⁻¹, 1575 cm⁻¹, and 1030 cm⁻¹ are assigned to O–H, C = O, C = C, and C–O stretching vibrations, respectively^31^. The relatively weak intensity of these peaks again indicates that functional groups are mainly located at the periphery of graphitic domains. Higher KOH ratios enhance surface oxidation and enrich the material with oxygen-containing functionalities, confirming the effectiveness of chemical activation. The introduction of these polar surface sites may enhance adsorption performance^27^.

In ZnCl₂-activated CDK samples, the structural vibrations at 1030 cm⁻¹ (C–O) and 1575 cm⁻¹ (C = C) remain clearly observable, while the broad band near 3300 cm⁻¹ (O–H) persists^32^. This suggests that complete graphitic ordering is not achieved under ZnCl₂ activation conditions. Compared with KOH activation, ZnCl₂ treatment yields a more cross-linked, rigid carbon network^33^.

As a result, comparing the AFDK and CDK series indicates that both precursors yield carbon materials with similar functional groups, while differences arise primarily in the degree of oxidation and surface functionalization, which are governed by the activating agent type and ratio.

### SEM analysis

**Fig. 4:**
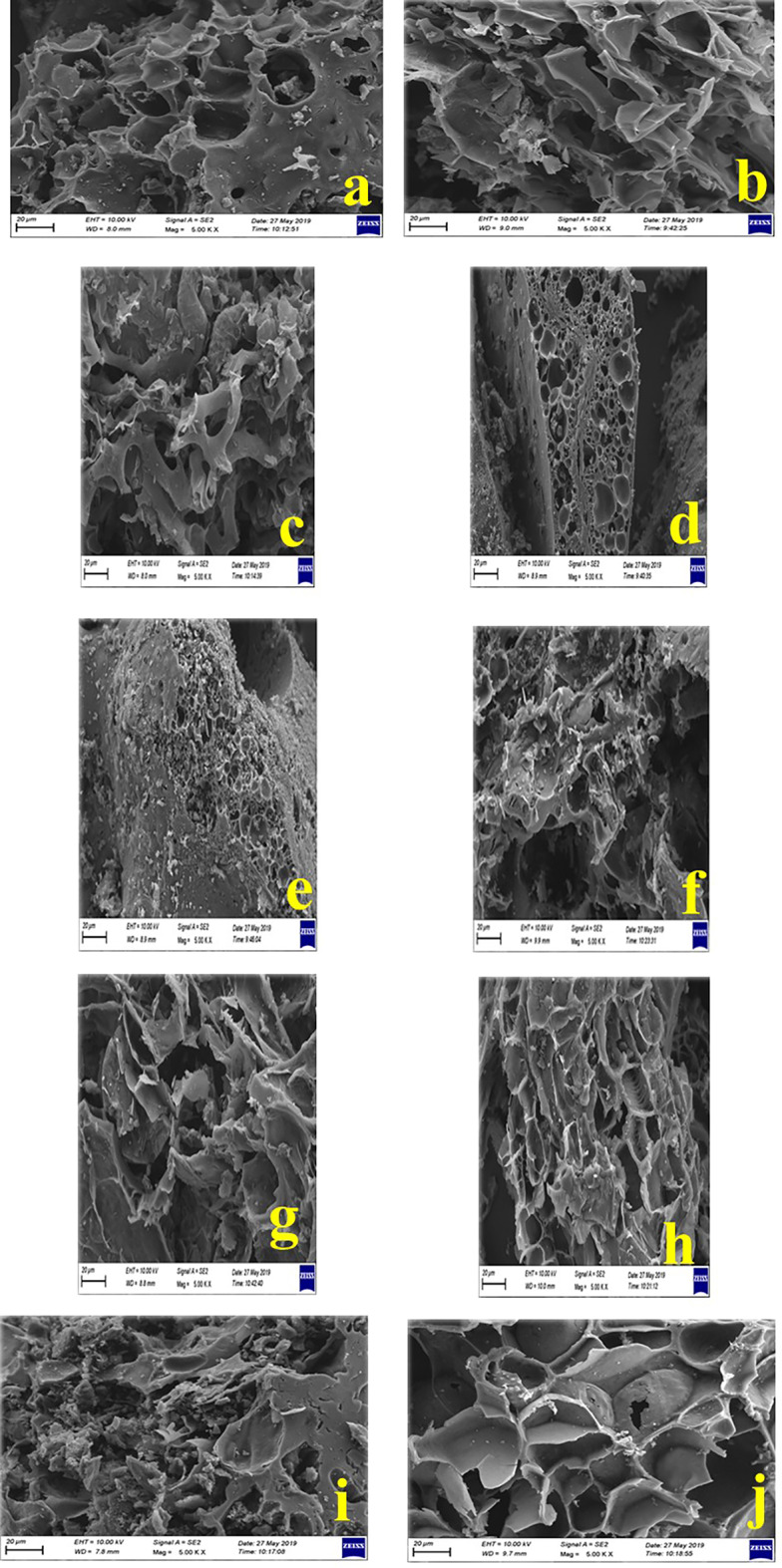
SEM micrographs of activated carbons prepared from pistachio and walnut shells at 5000× magnification: (**a**–**e**) AFDK series and (**f**–**j**) CDK series. (**a**) AFDK4, (**b**) AFDK5, (**c**) AFDK6, (**d**) AFDK9, (**e**) AFDK10, (**f**) CDK4, (**g**) CDK5, (**h**) CDK6, (**i**) CDK9, (**j**) CDK10.

The surface morphologies of the activated carbons derived from pistachio and walnut shells were examined by SEM at 5000× magnification (Figs. 4a–j). The images clearly demonstrate the influence of activation agent type (KOH or ZnCl₂), impregnation ratio, and mechanical grinding on pore development and surface topology. In the AFDK series, AFDK4 exhibits a moderately porous structure with irregular and non-uniform cavities, indicating limited structural degradation at lower KOH ratios. The relatively mild activation conditions led to partial breakdown of the macromolecular framework, yielding a restricted mesopore population and consequently a lower BET surface area.

As the KOH ratio increases (AFDK5 and AFDK6), the surface becomes progressively more fragmented. In AFDK5, lamellar and flake-like structures emerge, suggesting enhanced carbon rearrangement and the formation of graphenic domains. In AFDK6, a well-developed three-dimensional interconnected network is observed, characterized by lamellar sheets and irregular cylindrical voids. This hierarchical architecture correlates directly with the significant increase in surface area and mesopore volume, confirming that intensified KOH activation promotes deeper chemical etching and pore widening. In contrast, ZnCl₂-activated pistachio samples (AFDK9 and AFDK10) exhibit comparatively compact morphologies with shallow, bowl-shaped cavities. The absence of deep interconnected channels suggests that ZnCl₂ primarily induces dehydration and surface restructuring rather than aggressive pore expansion. The reduced homogeneity observed in AFDK10 indicates that grinding facilitates improved reagent penetration in ZnCl₂ systems; however, overall pore development remains limited compared to KOH activation.

Walnut-derived carbons (CDK series) display more pronounced pore evolution. CDK4 already shows ruptured pore walls and emerging cavities, indicating the onset of carbon framework decomposition. Increasing the KOH ratio (CDK5 and CDK6) leads to deeper cavities, larger voids, and highly interconnected channels. The stronger KOH etching of the lignocellulosic structure enhances pore volume and connectivity, generating a heterogeneous, rough surface that is favorable for adsorption processes^34^. The CDK6 sample exhibits the most developed hierarchical porous network, consistent with its highest measured BET surface area. ZnCl₂-activated walnut samples (CDK9 and CDK10) present rough surfaces with irregular pits formed primarily due to volatilization of organic components during thermal treatment. However, pore depth and interconnectivity are less pronounced than in KOH-activated samples. The similarity between ground and unground variants confirms that chemical activation dominates morphological development in ZnCl₂ systems.

Taken together, KOH activation produces more extensive pore formation, deeper cavities, and higher surface roughness than ZnCl₂ activation, particularly at higher impregnation ratios^35^. The SEM observations are fully consistent with BET and pore-size distribution analyses, demonstrating that increased KOH content promotes hierarchical porosity and mesopore formation, structural features critical for enhanced adsorption performance^36^.

### Elemental analysis

The elemental composition results (Table 2) provide quantitative evidence of the chemical transformations occurring during activation and clearly reflect the influence of both precursor type and activating agent on carbon enrichment and structural evolution. In the AFDK series, the carbon content increased dramatically from 45.54% in the raw precursor to over 90% in the highly KOH-activated samples (AFDK5 and AFDK6). This substantial increase indicates efficient removal of volatile components and heteroatoms, accompanied by progressive aromatic condensation and structural reorganization. The simultaneous decrease in oxygen content from 46.24% to approximately 7–9% confirms extensive deoxygenation, consistent with the intensified chemical etching mechanism of KOH activation. This deoxygenation process is accompanied by the reduction in oxygen-related FTIR bands and the emergence of more developed aromatic structures.

In contrast, ZnCl₂-activated AFDK samples retained higher oxygen contents (13–26%), suggesting a milder activation pathway dominated by dehydration and cross-linking rather than aggressive carbon gasification. The relatively preserved oxygen functionalities may contribute to increased surface polarity, although they are accompanied by lower surface area and limited structural rearrangement. A similar trend is observed in the CDK series. KOH activation increased carbon content from 40.48% in the raw walnut shell to approximately 87–89% in CDK4–CDK6, confirming efficient carbon enrichment. Compared to pistachio-derived samples, walnut-based carbons exhibit slightly lower final carbon percentages but demonstrate significantly higher surface areas, indicating that precursor macromolecular architecture plays a crucial role in pore evolution beyond elemental composition alone.

The lower hydrogen and nitrogen contents across highly activated samples further indicate progressive aromatization and the breakdown of lignocellulosic moieties^37^. Collectively, the elemental data confirm that KOH activation promotes deeper carbonization and structural ordering, while ZnCl₂ activation preserves a higher proportion of oxygen-containing functionalities, reflecting fundamentally different activation mechanisms. The variation in oxygen-containing functional groups observed with different activation strategies may significantly influence adsorption selectivity and surface–adsorbate interactions in practical applications. In particular, surface oxygen functionalities can contribute to electrostatic interactions, hydrogen bonding, and enhanced wettability in aqueous systems^31^.

**Table 2:** Results of elemental analysis of raw and activated carbons.

	C (% m/m)	H (% m/m)	*N* (% m/m)	S (% m/m)	O* (% m/m)
AFDK1	45.542	5.586	2.633	-	46.239
AFDK4	70.590	1.172	0.482	-	27.765
AFDK5	91.116	1.236	-	-	7.648
AFDK6	90.138	0.661	0.509	-	8.692
AFDK9	70.315	2.099	1.517	-	26.068
AFDK10	82.224	2.577	1.924	-	13.274
CDK1	40.479	4.703	1.022	-	53,796
CDK4	87.742	0.247	-	-	12.011
CDK5	88.545	0.265	-	-	11.190
CDK6	86.935	0.298	-	-	12.766
CDK9	80.138	1.941	1.215	-	16.311
CDK10	82.895	2.191	1.615	-	13.298

*Calculated from the difference.

### XRD analysis

**Fig. 5:**
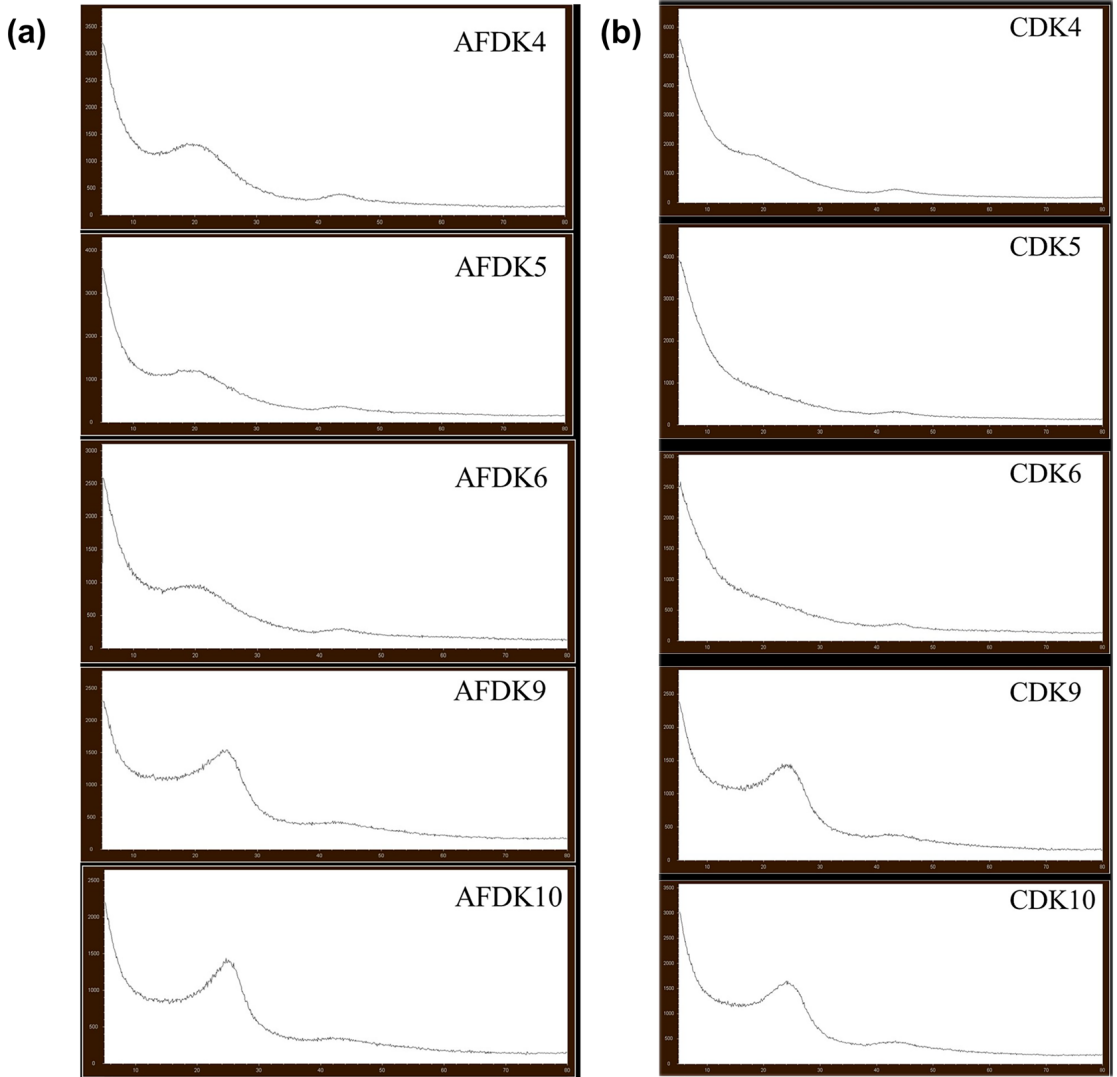
XRD spectra of (**a**) pistachio and (**b**) walnut shells.

The XRD patterns (Fig. 5) reveal the structural ordering and crystallinity of the activated carbons. All samples exhibit broad diffraction halos centered approximately at 2θ ≈ 20–25° and 43–45°, corresponding to the (002) and (100)/(101) reflections of turbostratic and disordered sp² carbon structures. The absence of sharp crystalline peaks confirms that the materials are predominantly amorphous. In the AFDK series, increasing the KOH ratio leads to broader and more intense halos, indicating enhanced structural disorder and the formation of defect-rich graphene-like domains. The progressive widening of the (002) reflection suggests reduced stacking order and increased interlayer spacing, consistent with the aggressive chemical etching and lattice expansion caused by KOH activation. These structural modifications are in agreement with the elevated surface areas and mesopore volumes observed in the BET analysis.

ZnCl₂-activated samples maintain amorphous characteristics but exhibit slightly more defined short-range ordering compared to highly KOH-treated carbons. This behavior suggests that ZnCl₂ promotes structural stabilization through cross-linking reactions rather than extensive lattice disruption. The persistence of broad peaks indicates incomplete graphitization, consistent with the FTIR results, which show retained oxygen functionalities. In the CDK series, walnut-derived carbons display a somewhat more pronounced (002) reflection compared to pistachio-derived samples, suggesting a slightly higher tendency toward local graphitic stacking. This observation may be attributed to the walnut shells’ inherent lignin-rich composition, which favors aromatic condensation during thermal treatment^25^. However, no crystalline inorganic residues are detected, confirming the effectiveness of post-activation washing.

Collectively, XRD analysis confirms that both activation routes yield predominantly amorphous, turbostratic carbon frameworks. KOH activation enhances structural disorder and defect density, correlating with higher porosity and surface area, whereas ZnCl₂ activation preserves partial short-range ordering and higher oxygen content. These structural distinctions are directly linked to the differences observed in textural properties and chemical composition.

## Conclusion

In this study, high-surface-area activated carbons were successfully synthesized from walnut green outer shells and pistachio pink outer shells via direct carbonization followed by chemical activation using KOH and ZnCl₂. The results clearly demonstrate that precursor composition and activation chemistry synergistically control pore evolution, structural disorder, and surface functionality. Among the investigated conditions, KOH activation at a 1:3 impregnation ratio produced the most developed textural properties, particularly for walnut-derived carbon (CDK6), which achieved an exceptionally high BET surface area of 2347 m² g⁻¹ along with a well-defined hierarchical pore network.

Compared with conventional coal-based and many biomass-derived activated carbons reported in the literature, the present approach offers a simple and effective strategy to obtain ultra-high-surface-area materials without complex pretreatment steps. The superior performance of walnut-derived carbons can be attributed to their lignin-rich composition, which promotes aromatic condensation and controlled pore widening during KOH activation, resulting in enhanced mesoporosity and interconnected channels favorable for mass transfer. In contrast, ZnCl₂ activation produced comparatively limited pore development under the selected conditions, highlighting the strong dependence of activation efficiency on reagent–biomass interactions.

From a sustainability perspective, the valorization of walnut and pistachio shell wastes provides a renewable alternative to fossil-based precursors. Nevertheless, similar to other KOH-based processes, high activation temperatures and chemical consumption remain important considerations for large-scale implementation, emphasizing the need for improved energy efficiency and chemical recovery strategies.

Owing to their ultra-high surface area, hierarchical pore architecture, and tunable surface chemistry, the synthesized carbons exhibit strong potential for adsorption-driven environmental applications, including water purification, dye removal, gas capture, and catalytic support systems. The structure–property relationships established in this work provide a rational framework for the targeted design of sustainable, high-performance porous carbons. Future research should focus on optimizing activation parameters, enhancing reagent recyclability, and validating performance in real wastewater systems to further advance practical applicability.

## Data Availability

The datasets used and/or analyzed during the current study are available from the corresponding author (Kadir Erol) upon reasonable request.
